# Prevalence of physical inactivity and associated factors among transgender people: a cross-sectional study, Baixada Santista, Brazil, 2023

**DOI:** 10.1590/S2237-96222026v35e20251283.en

**Published:** 2026-07-31

**Authors:** Katia Cristina Bassichetto, Margareth Guimarães Lima, Donatila Barbieri de Oliveira Souza, Lilian de Fatima Zanoni Nogueira, Carla Gianna Luppi, Barbara Iansã de Lima Barroso

**Affiliations:** 1 Faculdade de Ciências Médicas da Santa Casa de São Paulo SP São Paulo SP Brazil Faculdade de Ciências Médicas da Santa Casa de São Paulo, SP, Brazil; 2 Universidade Estadual de Campinas Faculdade de Ciências Médicas Campinas SP Brazil Universidade Estadual de Campinas, Faculdade de Ciências Médicas, Campinas, SP, Brazil; 3 Universidade de Sorocaba Sorocaba Sorocaba SP Brazil Universidade de Sorocaba, Sorocaba, SP, Brazil; 4 Universidade Federal de São Paulo São Paulo São Paulo SP Brazil Universidade Federal de São Paulo, São Paulo, SP, Brazil

**Keywords:** Physical Inactivity, Prevalence, Transgender Persons, Social Vulnerability, Cross-Sectional Studies, Conducta Sedentaria, Prevalencia, Personas Transgénero, Vulnerabilidad Social, Estudios Transversales

## Abstract

**Objective:**

To estimate prevalence of physical inactivity and investigate associated factors among self-identified transgender (trans) people.

**Methods:**

This was a cross-sectional study using data from the Baixada Santista Transgender Population Mapping survey. Trans people aged ≥18 years who lived, studied or worked that region in 2023 were recruited using a non-probabilistic sample. Those who reported not practicing physical activity were considered inactive. The independent variables were sociodemographic and variables related to well-being, described by relative and absolute frequencies, along with 95% confidence intervals (95%CI). In order to verify associations, Pearson’s chi-square test was applied with a 5% significance level, and logistic regression analyses with odds ratio (OR) estimation were performed.

**Results:**

Of the 237 participants, 40.9% were classified as inactive. The likelihood of leisure-time physical inactivity was higher among those with lower levels of education (OR 3.32; 95%CI 1.23; 8.94; p-value 0.018), those who had used mental health services in the last 12 months (OR 2.26; 95%CI 1.24; 4.09; p-value 0.007), and those with low quality of life (OR 2.39; 95%CI 1.32; 4.32; p-value 0.004). Physical inactivity was lower among people who experienced high levels of everyday discrimination (OR 0.45; 95%CI 0.21; 0.96; p-value 0.038).

**Conclusion:**

Physical inactivity in the population studied was associated with lower levels of education, use of mental health services and low quality of life. More active transgender people tended to suffer greater everyday discrimination, suggesting that adherence to physical activity is not only an individual choice but also a reflection of historical, social and environmental determinants.

## Resumen

**Objetivo:**

Estimar la prevalencia de inactividad física e investigar los factores asociados entre personas que se autoidentifican como transgénero.

**Métodos:**

Estudio transversal utilizando datos del Mapeo de la Población Transgénero de la Baixada Santista. Se reclutaron individuos mayores de 18 años que residían, estudiaban o trabajaban en esta región en 2023, mediante un muestreo no probabilístico. Se consideraron inactivos a quienes declararon no practicar actividad física. Las variables independientes fueron sociodemográficas y de bienestar, descritas mediante frecuencias relativas y absolutas, acompañadas de intervalos de confianza del 95% (IC95%). Para verificar las asociaciones, se aplicó la prueba chi-cuadrado de Pearson con un nivel de significancia del 5%, y se realizaron análisis de regresión logística con estimación de odds ratios (OR).

**Resultados:**

De los 237 participantes, el 40,9% se clasificó como inactivo. La probabilidad de inactividad física durante el tiempo libre fue mayor entre las personas con menor nivel educativo (OR 3,32; IC95 %: 1,23; 8,94; valor-p 0,018), las que habían utilizado servicios de salud mental en los últimos 12 meses (OR 2,26; IC95%: 1,24; 4,09; valor-p 0,007) y las que tenían una baja calidad de vida (OR 2,39; IC95%: 1,32; 4,32; valor-p 0,004). Entre las personas que experimentaron altos niveles de discriminación diaria, la inactividad física fue menor (OR 0,45; IC95%: 0,21; 0,96; valor-p 0,038).

**Conclusión:**

La inactividad física en la población estudiada se asoció con menores niveles de educación, uso de servicios de salud mental y baja calidad de vida. Las personas transgénero más activas tendían a sufrir mayor discriminación diaria, lo que sugiere que la adherencia a la actividad física no es solo una elección individual, sino también un reflejo de determinantes históricos, sociales y ambientales.

**Ethical aspects**

This research respected ethical principles, having obtained the following approval data:

Research ethics committee Secretaria de Estado da Saúde de São Paulo

Opinion number 6,242,582

Approval date 16/8/2023

Certificate of submission for ethical appraisal 64010722.8.0000.5375

Informed consent form Obtained from all participants prior to data collection.

## Introduction

Practicing physical activity is part of health prevention and promotion strategies, which is why it should be included in public policies aimed at the most vulnerable groups, in order to seek equity. The Global Action Plan on Physical Activity 2018-2030 provides guidelines for expanding actions, with a view to reducing physical inactivity by 15% by 2030 [1].

Recent data indicate that approximately one third of the world’s adult population does not meet the minimum recommendations for physical activity [2,3]. In Brazil, national health surveys, carried out with the general population, revealed high prevalence rates of leisure-time physical inactivity, namely, 70% in 2019 [4] and 63% in 2023 [5].

When considering the trans population, i.e. those whose gender identity differs from the sex assigned to them at birth [6], results from a systematic review [7] showed poorer indicators of physical and mental health and indicated that rates of physical inactivity in this population were consistently higher than in the cis population. 

A study conducted in the United Kingdom highlighted that psychological, bodily and social factors, such as transphobia, hinder trans people’s participation in physical activity practices, reflecting structural inequalities that cut across gender and identity. The study found that there are particularities among subgroups of the trans population [8]. Some, such as those using hormone therapy, showed greater body satisfaction and were more likely to increase their physical activity practices, which could promote mental well-being [8].

The trans population constitutes a group of marked heterogeneity, with life contexts that imply inequalities and differentiated risks regarding practices intended to promote health and prevent illness [9,10].

A scoping review of 46 Brazilian studies conducted with the trans population between 2019 and 2023 identified that in 67% of those studies, aspects related to social and/or programmatic vulnerability such as discrimination, situations of violence, unstable housing, prejudice, family exclusion, informal work and abusive use of alcohol and/or other drugs, among others – were present for the majority of this population, which demonstrated negative interference in access to health [9,11] and determined high prevalence rates of sexually transmitted infections, such as HIV [12], as well as mental health problems [11].

Cisnormativity, which is structural, is present in various institutions, including healthcare institutions, and confers privileges that naturalize being cisgender, pathologize being transgender, and deny trans people’s rights [13,10]. There is consistent criticism that transsexuality has been socially understood as an individual issue, failing to recognize transphobia as a social phenomenon that should be combated [10].

In Brazil, the persistence of scenarios of violence against the trans population, especially young Black trans women and transvestites (travestis), is reflected in the data on fatal violence and decreased life expectancy [14].

Physical inactivity, understood as a risk behavior and a product of discriminatory contexts, continues to be a priority outcome to be analyzed in research with transgender populations, especially in the Brazilian context, where there is little research on this topic.

The objective of this study was to estimate the prevalence of physical inactivity and to investigate potential associated factors among transgender people in the Baixada Santista region of São Paulo state in 2023.

## Methods

### Design

This is a cross-sectional study using data from the *São Paulo State Transgender Population Mapping survey: Baixada Santista Region *[15].

### Setting

In order to select potential study participants, we used an electronic pre-registration form, adapted from the form used by the São Paulo City Human Rights and Citizenship Department.

The participants were recruited using non-probability sampling. Data collection took place between August and December 2023.

### Participants

Eligible participants were those who self-identified as trans, travesti or non-binary, aged 18 or older, living, studying or working in one of the nine municipalities comprising the Baixada Santista region, and who answered positively to the question “Do you agree to be contacted to participate in a survey?”. They were contacted in order to schedule an interview, either in person or virtually, depending on availability. The online option was offered especially for individuals with mobility restrictions or psychosocial issues that made in-person attendance difficult, which helped ensure greater inclusion in the data collection process.

### Variables

The outcome of interest for this study was physical inactivity, a variable built by using the question: “In the last three months, have you practiced any type of physical activity or sport?” People who answered “no” were considered inactive. Those who answered that they practiced some type of physical activity, but did not reach the levels recommended by the World Health Organization (WHO), were classified as insufficiently active; while those who practiced at least 150 minutes of moderate physical activity or 75 minutes of vigorous physical activity were considered to be physically active (1).

### Data sources and measurement 

Physical activity practice, in an indoor or outdoor environment, and activity intensity variables were used to describe the sample, based on a list of physical activities presented in response to the question: “What is the main type of physical exercise or sport that you practice?”

The independent variables were demographic and socioeconomic: age group (18-29 years or ≥30 years); gender identity (trans woman/travesti, trans man/transmasculine, or non-binary person); race/skin color (White or Black – Black and mixed-race); schooling (incomplete high school or less, complete high school, complete bachelor’s degree, or complete bachelor’s degree and higher – for the logistic regression analysis, the complete high school and complete bachelor’s degree or higher categories were grouped); per capita income (up to 1 minimum wage, 1-3 minimum wages, or >3). 

Variables related to quality of life were analyzed using a five-question scale to assess well-being, with responses analyzed according to WHO recommendations [16], where 0 to 12 positive responses indicated good quality of life and 13 or more indicated poor quality of life. Mental health was also investigated through at least one positive response to the question: “Has any health professional informed you that you have any of these conditions?: anxiety; depression; substance dependence such as alcohol, tobacco and others; eating disorder; bipolar mood disorder; dysphoria; among others”, as well as self-assessment of mental health (excellent, very good, good, poor or very poor). Both variables were measured over the last two weeks. 

Everyday discrimination throughout life was analyzed, this being one of the dimensions that make up the Intersectional Discrimination Index [17], coded as 1 for yes versus 0 for no. Positive responses were added together for each of the following questions: “Because of who you are, have you ever: been treated as a hostile person, been offended, or have you seen/heard your identity being used as an insult?”; “Have you been treated as if others were afraid of you?”; “Have you been stared at or pointed at in public?”; “Have you been told that you should think, act, or look more like other people?”; “Have you heard that you, or people like you, shouldn’t be in certain places?”; “Have you been asked questions about inappropriate, offensive, or excessively intimate topics?”; “Have you been treated as if you were less intelligent or less capable than other people?”; “Have you been prevented from using or expelled from a public restroom?”. The responses generated an index categorized into slight, moderate and high levels of everyday discrimination based on the percentage distribution of the sample. Use of mental health services (yes or no) and presence of dysphoria (yes or no) were also investigated.

### Study size 

In order to determine the sample size, a value of 1.88% was applied to the total adult population of the municipalities comprising the Baixada Santista region, this being the only available national estimate of the magnitude of trans and non-binary people, obtained from a Brazilian estimate. A sample of 377 people was calculated, proportionally distributed between the municipalities of the aforementioned region, based on 19,965 people, a 5% margin of error and a design effect of 1 [15].

### Statistical methods

Descriptive analyses were performed on the demographic and socioeconomic characteristics (gender identity, race/skin color, schooling and per capita income) and physical activity practices of the trans population in the Baixada Santista region. The results are presented as absolute and relative frequencies, together with 95% confidence intervals (95%CI).

Pearson’s chi-square test was applied, with a 5% significance level, in order to verify associations between physical inactivity (dependent variable) and the independent variables. Logistic regression analyses were conducted, with estimation of odds ratios (OR) and their respective 95%CI.

The multiple logistic regression model was built using variables that had a p-value<0.200 in the preceding model. This included the variables of interest (sociodemographic, quality of life and mental health, everyday discrimination and dysphoria). A few variables were missing, and these were treated as missing data in the models (excluded from the analyses). Variables with a p-value<0.050 were kept in the final model, considered to be independently associated with the outcome (physical inactivity). All analyses were performed using Stata software, version 19.0.

## Results

The study sample consisted of 237 people. The majority were between 18 and 29 years old (64.1%), followed by 27.0% aged between 30 and 44 years and 8.9% aged ≥45 years. Regarding gender identity, 34.2% identified as trans women (n=80), 29.5% as trans men (n=69), 20.5% as non-binary persons (n=48), 8.6% as travestis (n=20) and 7.3% as transmasculine (n=17). As for race/skin color, 51.5% self-identified as White, 30.6% as mixed-race, and 17.9% as Black. In terms of schooling, 41.7% had completed a bachelor’s degree or higher, 38.8% had completed high school and 19.4% had completed elementary school or had incomplete high school education. Regarding monthly per capita income, 41.7% reported receiving up to ½ a minimum wage, 48.1% between ½ and 3 minimum wages and 10.2% more than 3 minimum wages. As for physical activity, 59.2% (95%CI 52.70; 65.29) of participants were considered active or insufficiently active (n=139).

Forty-nine percent were classified as inactive, as they did not practice any physical activity weekly (n=96). Among the physically active or insufficiently active individuals, 55.9% performed their activities indoors (n=71), and 44.1% outdoors (n=56). Regarding the intensity of the activities practiced, 23.7% performed mild physical activity (n=28), 52.5% moderate physical activity (n=62), and 23.7% vigorous physical activity (n=28) (Table 1).

**Table 1:** Number, percentage (%) and 95% confidence intervals (95%CI) of trans people demographic, socioeconomic and physical activity variables. Baixada Santista, 2023 (n=235)

Variable	n (%)	95%CI
**Age group **(years)	237 (100.0)	
18-29	152 (64.1)	57.8; 70.0
30-44	64 (27.0)	21.7; 33.1
≥45	21 (8.9)	5.8; 13.2
**Gender identity**	234 (100.0)	
Trans woman	80 (34.2)	28.4; 40.5
Trans man	69 (29.5)	24.0; 35.7
Non-binary person	48 (20.5)	15.8; 26.2
Travesti	20 (8.5)	5.6; 12.9
Transmasculine	17 (7.3)	4.5; 11.4
**Race/skin color**	235 (100.0)	
White	121 (51.5)	45.1; 57.9
Mixed-race	72 (30.6)	25.0; 36.9
Black	42 (17.9)	13.5; 23.3
Schooling	237 (100.0)	
Incomplete high school or less	46 (19.4)	14.8; 25.0
Complete high school	92 (38.8)	32.8; 45.2
Complete bachelor’s degree	61 (25.8)	20.5; 31.7
Higher education above bachelor’s degree	38 (16.0)	11.9; 21.3
**Monthly per capita income **(**minimum wages**)	235 (100.0)	
No income or <0.5	98 (41.7)	35.5; 48.2
0.5-3	113 (48.1)	41.7; 54.5
>3	24 (10.2)	6.9; 14.8
**Practices physical activity**	235 (100.0)	
Yes	139 (59.1)	52.7; 65.3
No	96 (40.9)	34.7; 47.3
**Environment where the physically active do their activities**	127 (100.0)	
Outdoor	56 (44.1)	35.6; 52.9
Indoor	71 (55.9)	47.1; 64.4
**Intensity of physically active activities**	118 (100.0)	
Mild	28 (23.7)	16.8; 32.4
Moderate	62 (52.6)	43.4; 61.5
Vigorous	28 (23.7)	16.8; 32.4

Table 2 shows the prevalence rates and unadjusted ORs for leisure-time physical inactivity among transgender and non-binary individuals, according to socioeconomic and demographic variables. Higher prevalence of physical inactivity was observed among people with lower levels of education: 58.7% among those with incomplete high school or less and 30% among those with high school education and a complete bachelor’s degree or higher. No statistically significant differences were found in the other variables analyzed. The results indicated that the likelihood of leisure-time physical inactivity was higher among less educated individuals (OR 3.49) (95%CI 1.40; 8.70) compared to those with a complete bachelor’s degree or higher.

**Table 2:** Number and prevalence (%) of leisure-time physical inactivity, according to socioeconomic and demographic variables, odds ratios (OR), 95% confidence intervals (95%CI) and p-values. Baixada Santista, 2023 (n=235)

Variable	n (%)	OR (95%CI)	p-value
**Age group **(years)	235		0.365
18-29	150 (38.7)	1.00	
≥30	85 (44.7)	1.28 (0.75; 2.20)	
**Gender identity **	232		0.401
Trans woman/travesti	100 (46.0)	1.60 (0.77; 3.29)	
Trans man/transmasculine	86 (39.5)	1.22 (0.58; 2.58)	
Non-binary	46 (34.8)	1.00	
**Race/skin color**	233		0.345
White	119 (38.7)	1.00	
Black (Black e mixed-race)	114 (44.9)	1.24 (0.76; 2.19)	
Schooling	235		0.006
Incomplete high school or less	46 (58.7)	2.47 (1.28; 4.77)	
High school and complete bachelor’s degree or above	189 (36.5)	1.00	
**Monthly income **(**minimum wages**)	233		0.759
No income or <0.5	98 (39.2)	1.08 (0.64; 1.85)	
≥0.5	137 (41.2)	1.00	

Table 3 shows, in the unadjusted analysis, higher prevalence of leisure-time physical inactivity among people who used mental health services in the last year (51.1%), compared to those who did not use these services (34.3%) (p-value 0.012), as well as among those who reported low quality of life (47.5%), compared to those who reported good quality of life (33.9%) (p-value 0.034).

**Table 3:** Number and prevalence (%) of leisure-time physical inactivity, according to quality of life and mental health indicators, odds ratios (OR), 95% confidence intervals (95%CI) and p-values. Baixada Santista, 2023 (n=235)

Variable	n (%)	OR (95%CI)	p-value
**Mental health diagnosis **	235		0.627
Present	79 (43.0)	1.15 (0.66; 1.98)	
Absent	156 (39.7)	1.00	
**Self-rated mental health**	234		0.066
Excellent to good	100 (34.0)	1.00	
Regular to very poor	134 (46.0)	1.65 (0.97; 2.81)	
**Everyday discrimination**	233		0.132
Slight	54 (44.4)	1.00	
Moderate	85 (47.1)	1.11 (0.56; 2.20)	
High	94 (33.0)	0.62 (0.30; 1.22)	
**Use of mental health service **	234		0.012
Yes	92 (51.1)	1.98 (1.16; 3.38)	
No	142 (34.5)	1.00	
**Presence of dysphoria**	235		0.668
Yes	27 (37.0)	0.83 (0.36; 1.91)	
No	208 (41.4)	1.00	
**Quality of life**	235		0.034
Poor	120 (47.5)	1.76 (1.04; 2.98)	
Good	115 (33.9)	1.00	

In the final multiple regression model (Table 4), the odds of leisure-time physical inactivity were higher among those with lower levels of education (OR 2.59; 95%CI 1.27; 5.29), among people who used mental health services in the last year (OR 2.31; 95%CI 1.28; 4.17), and among those with low quality of life (OR 2.42; 95%CI 1.34; 4.38). Physical inactivity was lower (OR 0.44; 95%CI 0.20; 0.93) among people who experienced high levels of everyday discrimination.

**Table 4:** Factors associated with leisure-time physical inactivity, adjusted odd ratiosa (OR), confidence intervals (95%CI) and p-values. Baixada Santista, 2023 (n=235)

Variable	Adjusted OR (95%CI)	p-value
Schooling		0.009
Incomplete high school or less	2.59 (1.27; 5.29)	
Complete high school or above	1.00	
**Use of health service**		0.005
Used	2.31 (1.28; 4.17)	
Did not use	1.00	
**Everyday discrimination **		
Slight	1.00	
Moderate	0.94 (0.45; 1.96)	0.865
High	0.44 (0.20; 0.93)	0.032
**Quality of life**		0.004
Low	2.42 (1.34; 4.38)	
High	1.00	

^a^Final multiple logistic regression model, OR adjusted for all the variables in the table.

The fit of the final model showed good calibration results between the observed and expected frequencies (Chi-square 4.69; p-value 0.583).

## Discussion

This study revealed that prevalence of leisure-time physical inactivity among those participating in the *Baixada Santista Transgender Population Mapping survey *[15] was lower than that of the general population in Brazil, but higher than that of transgender populations in international studies. The likelihood of physical inactivity was greater among people with less education, low quality of life, and those who used mental health services in the last 12 months. Conversely, half of those who experienced high levels of everyday discrimination were less likely to be physically inactive.

This study had limitations: scarcity of data to enable characterization of physical activity practices by to level of activity; the number of people included in the original study being lower than expected, despite being recruitment being widely publicized; and the methodology adopted for recruitment, i.e. use of a non-probabilistic sample. However, although it does not infer representativeness of the target population, the methodology is appropriate when dealing with a population that is difficult to access, where using probabilistic sampling is unfeasible.

It should be noted that the collection of self-reported data via internet through electronic registration may have excluded people without internet access, who are in a more vulnerable position. It is appropriate to mention the differences in the socioeconomic and demographic characteristics of the sample studied: more than 60% of the participants were young people (18-29 years old), and 80% had completed high school. This value is higher than that found in the first mapping of the trans population in the municipality of São Paulo (63.0%) [18]. These characteristics may be more representative of the population living in Santos, the main city in the Baixada Santista region, where 30% have completed a bachelor’s degree, according to data from the 2022 demographic census [19], a significantly higher proportion than the average for the Brazilian population [20]. It should be noted that the Baixada Santista region is privileged in that it concentrates a set of higher education institutions, which may have attracted trans people who may have migrated there with the aim of studying.

It should be highlighted that this study, by using data from a mapping that included people with heterogeneous gender identities, with a broad and diverse set of variables, made it possible to analyze association between physical inactivity and multiple factors, although it increased the possibility having to adjust for potential confounders. Even so, since this is a topic that has been little explored, including in the state of São Paulo and, especially, in Baixada Santista region, the findings provide initial parameters, which raise the possibility of further research and have expanded the available evidence base.

Given the specificities of the population studied and the geographical region in question, caution is recommended when directly comparing this study to similar studies with other populations, other geographical territories and other scenarios. As transgender people are frequently exposed to contexts of social exclusion and barriers to accessing healthcare, they remain invisible in comprehensive studies on healthy habits. This study, therefore, contributed to broadening the reflection on how structural inequalities can lead to increased physical inactivity and directly impact quality of life [21].

Given that the literature on physical activity among trans people is limited, we had to compare the findings of our study with those that involved only the general population, or with other studies that did include trans people, but with few participants.

The prevalence of physical inactivity found in this study (40.9%) was similar to that of the Latin American and Caribbean general population (41.2%) [3] and lower than that observed in a 2023 Brazilian national telephone health survey, also conducted with the general population (63.1%) [5], in addition to the level of physical activity found being insufficient in relation to what is recommended for reducing the risk of chronic non-communicable diseases and for improving health and well-being [1].

The association of higher prevalence of leisure-time physical inactivity among less educated people found in our study (58.7%) was also observed in a national survey, but in smaller proportions (40.4%) [5]. It is likely that more educated people have greater knowledge about health issues, better time management skills, more access to safe spaces for physical activity, more favorable social networks, and greater internalization of healthy living norms. Unlike the findings of this study, however, prevalence of physical inactivity in a survey mentioned earlier, conducted in Spain, was higher, both among those who completed higher education (74.4%) and among those who did not (77.8%) [23].

The difficulty faced by trans people in accessing and remaining in educational institutions has been characterized by these being violent spaces for this population, which hinders their continued enrollment and drives them to drop out [9,11]. The association between physical inactivity and low schooling, therefore, cannot be interpreted merely as a lack of knowledge or personal motivation, but rather as a result of a process of social exclusion that limits this population group’s access to safe spaces, leisure facilities and care practices.

The perception of low quality of life was also shown to be associated with greater odds of leisure-time physical inactivity, approximately twice as high compared to participants who reported high quality of life. A systematic review of 38 studies also found the same association among adults and older adults in the general population [24]. In contrast, a study conducted with trans people in a Brazilian state capital city found that quality of life was associated with engaging in physical activity among trans men [25].

The higher odds of physical inactivity observed among people who used mental health services (51.1%) compared to those who did not (34.5%) may be attributed to the direct impact of mental health disorders themselves. However, a similar association was observed in a survey conducted in Spain with transgender people, with higher prevalence rates. Those who self-reported mental health problems in the last year proportionally engaged in less physical activity (80.9% vs. 70.9%) [23]. It is clear that encouraging physical activity should be included as part of the guidance provided by mental health professionals.

Conversely, half of the participants who experienced high levels of everyday discrimination were less likely to be inactive. This association did not show statistical significance in the unadjusted analysis, but it did so after including the quality of life variable in the model, showing that discrimination is independently associated with physical activity, which can be explained by the greater exposure of more active people.

Visibility of their bodies is an aspect of the experiences of trans people, playing an ambivalent role in identity affirmation and daily life. The condition of being easily perceived as different exposes them to recurring situations of being scrutinized and microaggression [26]. At the same time, the need for a ‘convincing identity’, supported by a body that validates it, can mobilize body practices, including physical activity, as a strategy for building and affirming the body that is desired [20]. In this sense, it is possible that the relationship observed between higher levels of everyday discrimination and lower probability of physical inactivity reflects trajectories of bodily engagement oriented by the need for social recognition and identity validation. Previous studies [27,28] corroborate this interpretation by indicating that physical activity among trans people is associated with the need to modify physical appearance and, consequently, affirm their bodily identity.

These findings suggest that engagement in physical activities cannot be understood solely as an individual choice, but rather as a reflection of historical, social and environmental determinants that disproportionately affect vulnerable groups. It is important to prevent the burden of suffering arising from discrimination from falling on the individual and their gender expression. External gender recognition shapes exposure to discrimination and distributes safety and vulnerability differentially. This situation reflects a cisnormative system that structures physical activity spaces and practices based on binary gender patterns, which contributes to producing symbolic and material barriers to trans people’s participation in these spaces.

A literature review revealed that regular physical exercise among trans people has proven essential in mitigating violence against their bodies, as well as being important for the physical and mental health and well-being of this population [28]. Another aspect that has been problematized, mainly among trans men, refers to the possible interrelation between practicing physical activity and building their corporeality, which permeates social narratives and images that correspond to a specific expression of masculinity [29,30]. Muscle mass gain and definition resulting from physical activity is, for a segment of this population, part of the masculinization process that is being sought, which contributes to the construction of their identity [28].

To summarize, physical inactivity in the studied population was positively associated with lower levels of schooling, use of mental health services and poor quality of life. On the other hand, more active trans people tended to suffer greater everyday discrimination. Promoting physical activity in this population therefore demands actions that transcend the individual sphere and address the social inequities that permeate the daily lives of trans people.

Physical inactivity is a significant health risk factor and potentially modifiable, which is why it is important to investigate it in various population groups, such as trans people. It is important to understand transgender bodies as territories of dispute and resistance, which require health interventions formulated based on commitment to social justice and the right of all people to well-being.

There is also an urgent need to develop and improve culturally sensitive intersectoral public policies that consider the social determinants that impact health, in order to reduce the barriers faced by trans people. Inclusive environments and specific health promotion strategies should be implemented through intersectoral interventions, organized as part of a network and oriented towards equity.

## Data Availability

The database and the analysis codes used in this research are available upon request made to the authors.
