# Supplementary material for: Prevalence of physical inactivity and associated factors among transgender people: a cross-sectional study, Baixada Santista, Brazil, 2023
Source: Epidemiol Serv Saude. 2026 Jul 31;35:e20251283. doi: 10.1590/S2237-96222026v35e20251283.en (PMC13686307; doi:10.1590/S2237-96222026v35e20251283.en)
Supplement: Translation

# Translation: Prevalência de inatividade física e fatores associados entre pessoas trans: estudo transversal, Baixada Santista, 2023

**DOI:** 10.1590/S2237-96222026v35e20251283.pt

**Authors:** Katia Cristina Bassichetto, Margareth Guimarães Lima, Donatila Barbieri de Oliveira Souza, Lilian de Fatima Zanoni Nogueira, Carla Gianna Luppi, Barbara Iansã de Lima Barroso

**Affiliations:** 1 Faculdade de Ciências Médicas da Santa Casa de São Paulo SP São Paulo SP Brasil Faculdade de Ciências Médicas da Santa Casa de São Paulo, SP, Brasil; 2 Universidade Estadual de Campinas Faculdade de Ciências Médicas Campinas SP Brasil Universidade Estadual de Campinas, Faculdade de Ciências Médicas, Campinas, SP, Brasil; 3 Universidade de Sorocaba Sorocaba Sorocaba SP Brasil Universidade de Sorocaba, Sorocaba, SP, Brasil; 4 Universidade Federal de São Paulo São Paulo São Paulo SP Brasil Universidade Federal de São Paulo, São Paulo, SP, Brasil

**Keywords:** Inatividade Física, Prevalência, Pessoas Transgênero, Vulnerabilidade Social, Estudos Transversais

## Abstract

**Objetivo:**

Estimar a prevalência de inatividade física e investigar fatores associados entre pessoas autodeclaradas trans.

**Métodos:**

Estudo transversal com dados do Mapeamento da População Trans da Baixada Santista. Foram recrutadas, por amostra não probabilística, pessoas com ≥18 anos que residiam, estudavam ou trabalhavam nessa região em 2023. Consideraram-se inativas as que declararam não praticar atividade física. As variáveis independentes foram sociodemográficas e de bem-estar, descritas por frequências relativas e absolutas, acompanhadas dos intervalos de confiança de 95% (IC95%). Para verificar associações, aplicou-se o teste do qui-quadrado de Pearson com nível de significância de 5% e foram conduzidas análises de regressão logística com estimativa de *odds ratios* (OR).

**Resultados:**

Das 237 pessoas participantes, 40,9% foram classificadas como inativas. A chance de inatividade física no lazer foi maior entre as menos escolarizadas (OR 3,32; IC95% 1,23; 8,94; p-valor 0,018), as que utilizaram serviço de saúde mental nos últimos 12 meses (OR 2,26; IC95% 1,24; 4,09; p-valor 0,007) e as que apresentaram baixa qualidade de vida (OR 2,39; IC95% 1,32; 4,32; p-valor 0,004). Entre as pessoas que vivenciaram alto nível de discriminação cotidiana, a inatividade física foi menor (OR 0,45; IC95% 0,21; 0,96; p-valor 0,038).

**Conclusão:**

A inatividade física na população estudada esteve associada ao menor nível de escolaridade, ao uso de serviço de saúde mental e à baixa qualidade de vida. As pessoas trans mais ativas tenderam a sofrer maior discriminação cotidiana, o que sugeriu que a adesão à atividade física é não só uma escolha individual, mas um reflexo de determinações históricas, sociais e ambientais.

**Aspectos éticos**

Esta pesquisa respeitou os princípios éticos, obtendo os seguintes dados de aprovação:

Comitê de ética em pesquisa Secretaria de Estado da Saúde de São Paulo

Número do parecer 6.242.582

Data de aprovação 16/8/2023

Certificado de apresentação de apreciação ética 64010722.8.0000.5375

Registro do consentimento livre e esclarecido Obtido de todas as pessoas participantes antes da coleta dos dados.

## Introdução

A prática de atividade física compõe as estratégias de prevenção e de promoção da saúde, motivo pela qual devem ser parte das políticas públicas direcionadas aos grupos mais vulnerabilizados, a fim de buscar equidade. O Plano de Ação Global para a Atividade Física 2018-2030 forneceu orientações para ampliação de ações, com vistas à redução da inatividade física em 15% até 2030 [1]. 

Dados recentes apontam que aproximadamente um terço da população adulta mundial não cumpre as recomendações mínimas de atividade física [2,3]. No Brasil, inquéritos nacionais de saúde, realizados com a população geral, revelaram altas prevalências de inatividade física no lazer, de 70% em 2019 [4] e de 63% em 2023 [5].

Ao considerar a população trans, aquela cuja identidade de gênero diverge do sexo atribuído ao nascimento [6], resultados de uma revisão sistemática [7] mostraram piores indicadores de saúde física e mental e indicaram que os índices de inatividade física nessa população eram consistentemente mais altos do que na população cis. 

Estudo desenvolvido no Reino Unido reforçou que fatores psicológicos, corporais e sociais, como transfobia, dificultam a participação de pessoas trans em práticas de atividade física, o que reflete desigualdades estruturais que atravessam o gênero e a identidade. Foi verificado que há particularidades entre subgrupos da população trans [8]. Alguns, como aqueles em uso de hormonização, apresentaram maior satisfação corporal e estiveram mais propensos a aumentar a prática de atividade física, o que poderia propiciar bem-estar mental [8].

A população trans constitui um grupo de marcante heterogeneidade, com contextos de vida que implicam em desigualdades e em riscos diferenciados de práticas para promoção da saúde e prevenção de adoecimentos [9,10].

Uma revisão de escopo de 46 estudos brasileiros feitos com população trans, entre 2019 e 2023, identificou que em 67% desses, aspectos relacionados à vulnerabilidade social e/ou programática como discriminação, situações de violência, moradia instável, preconceito, exclusão familiar, trabalho informal e uso abusivo de álcool e/ou outras drogas, entre outros – estiveram presentes para a maioria dessa população, o que demonstrou interferir negativamente no acesso à saúde [9,11] e determinou altas prevalências de infecções sexualmente transmissíveis, a exemplo do HIV [12] e de agravos à saúde mental [11]. 

A cisnormatividade, por ser estrutural, se faz presente em diversas instituições, inclusive na saúde, e confere privilégios que naturalizam a cisgeneridade, patologizam as transgeneridades e negam seus direitos [13,10]. Há uma crítica consistente de que as transexualidades têm sido compreendidas socialmente como uma questão do indivíduo, não reconhecendo a transfobia como um fenômeno social a ser combatido [10]. 

No Brasil, a persistência de cenários de violência contra a população trans, em especial de mulheres trans e de travestis jovens e negras, se refletem nos dados de violência letal e de diminuição da expectativa de vida [14]. 

A inatividade física, por sua vez, compreendida como comportamento de risco e produto de contextos discriminatórios, continua sendo um desfecho prioritário a ser analisado em pesquisas com populações trans, ainda mais no cenário brasileiro, onde há poucas pesquisas com essa temática. 

Este estudo tem por objetivo estimar a prevalência de inatividade física e investigar potenciais fatores associados entre pessoas trans da Baixada Santista, região do estado de São Paulo, em 2023. 

## Métodos

### Desenho do estudo

Estudo transversal com a utilização de dados do *Mapeamento da População Trans do Estado de São Paulo: Região da Baixada Santista *[15].

### Contexto

Para seleção das potenciais pessoas participantes do estudo, foi utilizado um pré-cadastro eletrônico, adaptado de formulário da Secretaria Municipal de Direitos Humanos e Cidadania de São Paulo. 

As pessoas participantes foram recrutadas por meio de amostra não probabilística, e a coleta de dados ocorreu no período de agosto a dezembro de 2023.

### Participantes

Foram consideradas elegíveis as pessoas autodeclaradas trans, travestis ou não binárias com ≥18 anos que residiam, estudavam ou trabalhavam em um dos nove municípios da região da Baixada Santista e que responderam positivamente à pergunta “Você aceita que entremos em contato para participar de uma pesquisa?”. Elas foram contatadas para agendamento da entrevista, presencial ou virtual, conforme disponibilidade. A modalidade virtual foi oferecida especialmente para pessoas com restrições de mobilidade ou questões psicossociais que dificultassem o comparecimento presencial, o que ajudou a assegurar maior inclusão na coleta de dados. 

### Variáveis

O desfecho de interesse para este estudo foi a inatividade física, variável construída por meio da questão: “Nos últimos três meses, praticou algum tipo de atividade física ou esporte?”. Foram consideradas inativas as pessoas que assinalaram a resposta “não”. As que responderam que praticavam alguma atividade física, mas não atingiram os níveis recomendados pela Organização Mundial de Saúde (OMS), foram classificadas como insuficientemente ativas; e ativas, as que praticavam pelo menos 150 minutos de atividade física moderada ou 75 de atividade física intensa (1).

### Fontes de dados e Mensuração 

Para a descrição da amostra, foram utilizadas as variáveis de prática de atividade física, em ambiente fechado ou aberto, e de intensidade da atividade, a partir da apresentação de uma lista de atividades físicas relacionada à pergunta: “Qual o tipo principal de exercício físico ou esporte que você pratica?”. 

As variáveis independentes foram as demográficas e as socioeconômicas: faixa etária (18-29 anos ou ≥30 anos); identidade de gênero (mulher trans/travesti, homem trans/transmasculino ou pessoa não binária); raça/cor da pele (branca ou negra – pretos e pardos); escolaridade (ensino médio incompleto ou menos, ensino médio completo, superior completo ou superior completo ou mais – para a análise de regressão logística, as categorias ensino médio e superior completo ou mais foram agrupadas); renda per capita (até 1 salário mínimo, de 1-3 salários mínimos ou >3). 

Foram analisadas variáveis relativas à qualidade de vida, por meio da aplicação de escala com cinco questões para avaliar o bem-estar, cujas respostas foram analisadas conforme recomendação da OMS [16], em que 0 a 12 respostas positivas indicavam qualidade de vida boa e 13 ou mais apontavam para qualidade de vida ruim. A saúde mental também foi investigada, por meio de pelo menos uma resposta positiva à questão: “Algum(a) profissional te informou que você tem alguma dessas condições?: ansiedade; depressão; dependência de uso de substâncias como álcool, tabaco e outras; transtorno alimentar; transtorno de humor bipolar; disforia; entre outros”, bem como a autoavaliação da saúde mental (excelente, muito boa, boa, ruim ou muito ruim). Ambas as variáveis foram medidas quanto às duas últimas semanas. 

Foi analisada a discriminação cotidiana ao longo da vida, uma das dimensões que compõem o Índice de Discriminação Interseccional [17], codificada como 1 para sim versus 0 para não. Foram somadas as respostas positivas para cada uma das questões: “Por ser quem você é, alguma vez: te trataram como uma pessoa hostil, te ofenderam ou você viu/ouviu sua identidade sendo usada como um insulto?”; “Te trataram como se os outros tivessem medo de você?”; Te observaram ou apontaram em público?”; “Te falaram que deveria pensar, agir ou se parecer mais com outras pessoas?”; “Ouviu que você, ou pessoas como você, não deveriam estar em um determinado local?”; “Te fizeram perguntas sobre assuntos inapropriados, ofensivos ou excessivamente íntimos?”; “Te trataram como se fosse menos inteligente ou capaz do que outras pessoas?”; “Te impediram de usar ou expulsaram de um banheiro público?”. As respostas geraram um índice categorizado em discriminação cotidiana leve, mediana e alta baseado na distribuição percentual da amostra. Também foi investigado o uso de serviço de saúde mental (sim ou não) e a presença de disforia (sim ou não).

### Tamanho do estudo 

Para determinar o tamanho amostral, foi aplicado o valor de 1,88% sobre o total da população adulta dos municípios pertencentes à Baixada Santista, única estimativa nacional disponível da magnitude de pessoas trans e não-binárias, obtida a partir de uma estimativa brasileira. Calculou-se uma amostra de 377 pessoas, proporcionalmente distribuída entre os municípios da supracitada região, com base em 19.965 pessoas, margem de erro de 5% e efeito de desenho de 1 [15]. 

### Métodos estatísticos

Foram realizadas análises descritivas das características demográficas e socioeconômicas (identidade de gênero, raça/cor de pele, escolaridade e renda per capita) e da prática de atividade física da população trans da Baixada Santista. Os resultados foram apresentados em frequências absolutas e relativas, acompanhados dos intervalos de confiança de 95% (IC95%).

Para verificar associações entre a inatividade física (variável dependente) e as variáveis independentes, foi aplicado o teste do qui-quadrado de Pearson, com nível de significância de 5%. Foram conduzidas análises de regressão logística, com estimativa das razões de chance (*odds ratio*, OR) e seus respectivos IC95%.

Para compor o modelo múltiplo de regressão logística, foram selecionadas as variáveis que apresentaram p-valor<0,200 no modelo anterior. Esse incluiu as variáveis de interesse (sociodemográficas, qualidade de vida e saúde mental, discriminação cotidiana e disforia). Foram poucas as informações não preenchidas em algumas variáveis, tratadas depois como dados faltantes nos modelos (excluídas das análises). Permaneceram no modelo final aquelas com p-valor<0,050, consideradas associadas de forma independente ao desfecho (inatividade física). Todas as análises foram realizadas no software Stata, versão 19.0.

## Resultados

A amostra do estudo foi composta por 237 pessoas. A maioria tinha entre 18 e 29 anos (64,1%), seguida por 27,0% com idades entre 30 e 44 anos e por 8,9% com ≥45 anos. Em relação à identidade de gênero, 34,2% se identificaram como mulher trans (n=80), 29,5% como homem trans (n=69), 20,5% como pessoa não binária (n=48), 8,6% como travesti (n=20) e 7,3% como transmasculino (n=17). Quanto à raça/cor da pele, 51,5% se autodeclararam pessoas brancas, 30,6% pardas e 17,9% pretas. Em termos de escolaridade, 41,7% tinham ensino superior completo ou mais, 38,8% tinham ensino médio completo e 19,4% tinham ensino fundamental completo ou médio incompleto. Em relação à renda per capita mensal, 41,7% declararam receber até 1/2 salário mínimo, 48,1% entre 1/2 e 3 salários mínimos e 10,2% acima de 3 salários mínimos. Quanto à prática de atividade física, 59,2% (IC95% 52,70; 65,^29^) das pessoas participantes foram considerados ativas ou insuficientemente ativas (n=139). 

Foram classificados como pessoas inativas 40,9%, por não praticarem qualquer atividade física semanalmente (n=96). Entre as pessoas fisicamente ativas ou insuficientemente ativas, 55,9% realizavam suas atividades em ambientes fechados (n=71), e 44,1% em ambientes abertos (n=56). Em relação à intensidade da prática, 23,7% realizavam atividade física leve (n=28), 52,5% atividade física moderada (n=62) e 23,7% atividade física vigorosa (n=28) (Tabela 1).

**Tabela 1:** Número, porcentagem (%) e intervalos de confiança de 95% (IC95%) de variáveis demográficas, socioeconômicas e de atividade física da população trans. Baixada Santista, 2023 (n=235)

Variável	n (%)	IC95%
**Faixa etária **(anos)	237 (100,0)	
18-29	152 (64,1)	57,8; 70,0
30-44	64 (27,0)	21,7; 33,1
≥45	21 (8,9)	5,8; 13,2
**Identidade de gênero**	234 (100,0)	
Mulher trans	80 (34,2)	28,4; 40,5
Homem trans	69 (29,5)	24,0; 35,7
Pessoa não binária	48 (20,5)	15,8; 26,2
Travesti	20 (8,5)	5,6; 12,9
Transmasculino	17 (7,3)	4,5; 11,4
**Raça/cor da pele**	235 (100,0)	
Branca	121 (51,5)	45,1; 57,9
Parda	72 (30,6)	25,0; 36,9
Preta	42 (17,9)	13,5; 23,3
Escolaridade	237 (100,0)	
Médio incompleto ou menos	46 (19,4)	14,8; 25,0
Médio completo	92 (38,8)	32,8; 45,2
Superior completo	61 (25,8)	20,5; 31,7
Maior que superior completo	38 (16,0)	11,9; 21,3
**Renda per capita mensal **(**salários mínimos**)	235 (100,0)	
Sem renda ou <0,5	98 (41,7)	35,5; 48,2
0,5-3	113 (48,1)	41,7; 54,5
>3	24 (10,2)	6,9; 14,8
**Prática de atividade física**	235 (100,0)	
Sim	139 (59,1)	52,7; 65,3
Não	96 (40,9)	34,7; 47,3
**Ambiente da prática entre os ativos**	127 (100,0)	
Aberto	56 (44,1)	35,6; 52,9
Fechado	71 (55,9)	47,1; 64,4
**Intensidade da prática entre os ativos**	118 (100,0)	
Leve	28 (23,7)	16,8; 32,4
Moderada	62 (52,6)	43,4; 61,5
Vigorosa	28 (23,7)	16,8; 32,4

A Tabela 2 apresentou as prevalências e os OR brutos de inatividade física no lazer entre pessoas trans e não binárias, segundo variáveis socioeconômicas e demográficas. Observou-se maior prevalência de inatividade física entre pessoas com menor escolaridade: 58,7% entre aquelas com ensino médio incompleto ou menos e 30% entre as com ensino médio e superior completo ou mais. Não foram encontradas diferenças estatisticamente significativas nas demais variáveis analisadas. Os resultados apontaram que a chance de inatividade física no lazer foi maior entre as pessoas menos escolarizadas (OR 3,49) (IC95% 1,40; 8,70) em comparação àquelas com superior completo ou mais. 

**Tabela 2:** Número e prevalência (%) de inatividade física no lazer, segundo variáveis socioeconômicas e demográficas, razão de chances (*odds ratio*, OR), intervalo de confiança de 95% (IC95%) e p-valor. Baixada Santista, 2023 (n=235)

Variável	n (%)	OR (IC95%)	p-valor
**Faixa etária **(anos)	235		0,365
18-29	150 (38,7)	1,00	
≥30	85 (44,7)	1,28 (0,75; 2,20)	
**Identidade de gênero **	232		0,401
Mulher trans/travesti	100 (46,0)	1,60 (0,77; 3,29)	
Homem trans/transmasculino	86 (39,5)	1,22 (0,58; 2,58)	
Não binária	46 (34,8)	1,00	
**Raça/cor da pele**	233		0,345
Branca	119 (38,7)	1,00	
Negra (preta e parda)	114 (44,9)	1,24 (0,76; 2,19)	
Escolaridade	235		0,006
Ensino médio incompleto ou menos	46 (58,7)	2,47 (1,28; 4,77)	
Ensino médio e superior completo ou mais	189 (36,5)	1,00	
**Renda mensal **(**salários mínimos**)	233		0,759
Sem renda ou <0,5	98 (39,2)	1,08 (0,64; 1,85)	
≥0,5	137 (41,2)	1,00	

A Tabela 3 apresentou, na análise bruta, maior prevalência de inatividade física no lazer entre as pessoas que utilizaram serviços de saúde mental no último ano (51,1%), em comparação àquelas que não utilizaram esses serviços (34,3%) (p-valor 0,012), bem como entre as que relataram uma baixa qualidade de vida (47,5%), em relação às que relataram boa qualidade de vida (33,9%) (p-valor 0,034). 

**Tabela 3:** Número e prevalência (%) de inatividade física no lazer, segundo indicadores de qualidade de vida e de saúde mental, razão de chances (*odds ratio*, OR), intervalo de confiança de 95% (IC95%) e p-valor. Baixada Santista, 2023 (n=235)

Variável	n (%)	OR (IC95%)	p-valor
**Diagnóstico de saúde mental**	235		0,627
Presente	79 (43,0)	1,15 (0,66; 1,98)	
Ausente	156 (39,7)	1,00	
**Autoavaliação de saúde mental**	234		0,066
Excelente a boa	100 (34,0)	1,00	
Regular a muito ruim	134 (46,0)	1,65 (0,97; 2,81)	
**Discriminação cotidiana**	233		0,132
Leve	54 (44,4)	1,00	
Mediana	85 (47,1)	1,11 (0,56; 2,20)	
Alta	94 (33,0)	0,62 (0,30; 1,22)	
**Uso de serviço de saúde mental**	234		0,012
Sim	92 (51,1)	1,98 (1,16; 3,38)	
Não	142 (34,5)	1,00	
**Presença de disforia**	235		0,668
Sim	27 (37,0)	0,83 (0,36; 1,91)	
Não	208 (41,4)	1,00	
**Qualidade de vida**	235		0,034
Ruim	120 (47,5)	1,76 (1,04; 2,98)	
Boa	115 (33,9)	1,00	

No modelo final de regressão múltipla (Tabela 4), a chance de inatividade física no lazer foi maior entre os menos escolarizados (OR 2,59; IC95% 1,27; 5,^29^), entre as pessoas que usaram serviço de saúde mental no último ano (OR 2,31; IC95% 1,28; 4,^17^) e entre as que apresentaram qualidade de vida baixa (OR 2,42; IC95% 1,34; 4,38). Entre as pessoas que vivenciaram alto nível de discriminação cotidiana, a inatividade física foi menor (OR 0,44; IC95% 0,20; 0,93).

**Tabela 4:** Fatores associados à inatividade física no lazer, razão de chances (*odds ratio*, OR) ajustadas^a^, intervalos de confiança (IC95%) e p-valor. Baixada Santista, 2023 (n=235)

Variável	OR ajustada (IC95%)	p-valor
Escolaridade		0,009
Médio incompleto ou menos	2,59 (1,27; 5,29)	
Médio completo ou mais	1,00	
**Uso de serviço de saúde**		0,005
Utilizou	2,31 (1,28; 4,17)	
Não utilizou	1,00	
**Discriminação cotidiana**		
Leve	1,00	
Mediana	0,94 (0,45; 1,96)	0,865
Alta	0,44 (0,20; 0,93)	0,032
**Qualidade de vida**		0,004
Baixa	2,42 (1,34; 4,38)	
Alta	1,00	

^a^Modelo múltiplo final de regressão logística, OR ajustados por todas as variáveis da tabela.

A adequação do modelo final apresentou resultados de boa calibração entre as frequências observadas e esperadas (Qui-quadrado 4,69; p-valor 0,583). 

## Discussão

O presente estudo revelou que a prevalência de inatividade física no lazer das pessoas participantes do Mapeamento da População Trans da Baixada Santista [15] foi inferior a da população geral no Brasil, porém superior a de populações trans de estudos internacionais. A chance de inatividade física foi maior entre as pessoas menos escolarizadas, com baixa qualidade de vida e que utilizaram serviços de saúde mental nos últimos 12 meses. Em contrapartida, metade das que vivenciaram alto nível de discriminação cotidiana tiveram menor probabilidade de serem inativas. 

Este estudo apresentou limitações: a escassez de dados para caracterizar a prática de atividade física segundo níveis; o número de pessoas incluídas no estudo original ter ficado abaixo do esperado, apesar da divulgação exaustiva; a metodologia adotada para o recrutamento – o uso de amostra não probabilística. Porém, apesar de não inferir representatividade da população-alvo, ela é apropriada quando se trata de população de difícil acesso, com inviabilidade de aplicação de amostragem probabilística.

Ressalta-se que a coleta de dados autorreferidos por internet a partir de cadastro eletrônico pode ter excluído pessoas sem acesso à rede, em maior condição de vulnerabilização. Cabe mencionar a diferenciação das características socioeconômicas e demográficas da amostra estudada: mais de 60% das pessoas participantes eram jovens (18-29 anos), e 80% tinham completado o ensino médio. Esse valor é superior ao verificado no primeiro mapeamento da população trans do município de São Paulo (63,0%) [18]. Tais características podem ser mais representativas da população residente em Santos, principal cidade da região, onde 30% tem ensino superior completo, segundo dados do censo demográfico de 2022 [19], proporção significativamente maior que a média da população brasileira [20]. Vale considerar que a região da Baixada Santista é privilegiada por concentrar um conjunto de instituições de ensino superior, o que pode ter atraído pessoas trans, que migraram com o objetivo de estudar. 

Cabe ressaltar que o presente estudo, por utilizar dados de um mapeamento que incluiu pessoas com identidades de gênero heterogêneas, com um conjunto amplo e diversificado de variáveis, possibilitou analisar a associação entre inatividade física e múltiplos fatores, embora tenha aumentado a possibilidade de ajuste para potenciais confundidores. Ainda assim, por se tratar de um tema pouco explorado, inclusive no estado de São Paulo e, em especial, nessa região, os achados ofereceram parâmetros iniciais, o que suscitou a possibilidade de novas pesquisas e ampliou a base de evidências disponíveis. 

Dadas as especificidades da população estudada e da região geográfica em questão, recomenda-se cautela na comparação direta a estudos semelhantes com outras populações, outros territórios geográficos e outros cenários. Como pessoas trans estão frequentemente expostas a contextos de exclusão social e a barreiras no acesso à saúde, elas permanecem invisibilizadas em estudos abrangentes sobre hábitos saudáveis. Este estudo, então, contribuiu para ampliar a reflexão sobre como desigualdades estruturais podem levar ao aumento da inatividade física e impactar diretamente na qualidade de vida [21]. 

Já que a literatura é limitada sobre atividade física em pessoas trans, foi necessário comparar os achados do presente estudo com os que envolveram somente a população geral, ou com outros que incluíram a população trans, porém em número reduzido. 

A prevalência de inatividade física encontrada neste estudo (40,9%) foi semelhante à da população geral da América Latina e do Caribe (41,2%) [3] e inferior à observada em inquérito telefônico nacional de saúde de 2023, também realizado com a população geral (63,1%) [5], além de se mostrar insuficiente ao recomendado para redução do risco de doenças crônicas não transmissíveis e para melhora da saúde e do bem-estar [1]. 

Em inquéritos online realizados nos EUA com população trans, observou-se semelhança com os valores da população transfeminina (43,3%) e índices maiores do que os da população transmasculina (31,0%) [22]. Já na Espanha, a prevalência de inatividade física na população trans estudada (76,8%) foi maior do que a do presente estudo [23].

A associação de maior prevalência de inatividade física no lazer entre as pessoas menos escolarizadas verificada no presente estudo (58,7%) também foi observada em inquérito nacional, porém em menores proporções (40,4%) [5]. É provável que pessoas mais escolarizadas tenham maior conhecimento sobre questões de saúde, maiores condições de planejar o tempo, mais acesso a espaços seguros para a prática de atividade física, redes sociais mais favoráveis e maior internalização de normas de vida saudável. Diferentemente dos achados deste estudo, no entanto, a prevalência de inatividade física em inquérito já mencionado, realizado na Espanha, foi maior, tanto entre os que concluíram o ensino superior (74,4%) quanto entre os que não concluíram (77,8%) [23].

A dificuldade da população trans no acesso e permanência a instituições educacionais tem se caracterizado na configuração delas como espaços violentos para essa população, o que dificulta-lhes a permanência e impulsiona-lhes a evasão [9,11]. A associação entre inatividade física e baixa escolaridade, portanto, não pode ser interpretada apenas como falta de conhecimento ou de incentivo pessoal, mas como resultado de um processo de exclusão social que limita o acesso desse grupo populacional a espaços seguros, a equipamentos de lazer e a práticas de cuidado. 

A percepção de baixa qualidade de vida também se mostrou associada à maior chance de inatividade física do lazer, aproximadamente o dobro em comparação às pessoas que referiram alta qualidade de vida. Em revisão sistemática com 38 estudos também foi observada essa mesma associação em adultos e em idosos da população geral [24]. Em contrapartida, em estudo realizado com pessoas trans em uma capital brasileira, foi verificado entre os homens que a qualidade de vida estava associada à prática de atividade física [25].

A maior chance de inatividade física observada entre as pessoas que utilizaram serviços de saúde mental (51,1%) em relação às que não utilizaram (34,5%) pode ser atribuída ao impacto direto dos próprios transtornos. Em inquérito realizado na Espanha com pessoas trans, porém, associação semelhante foi observada, com prevalências mais elevadas. As que autorreferiram problemas de saúde mental no último ano, praticavam proporcionalmente menos atividade física (80,9% vs. 70,9%) [23]. Percebe-se que o incentivo à prática de atividade física deve constar do rol de orientações dos profissionais de saúde mental. 

Em contrapartida, a metade das pessoas participantes que vivenciaram alto nível de discriminação cotidiana, tiveram menor probabilidade de serem inativas. Essa associação não apresentou significância estatística na análise bruta, mas sim após inserção da variável qualidade de vida no modelo, mostrando que a discriminação se associa de maneira independente à prática de atividade física, o que pode ser explicado pela maior exposição das pessoas mais ativas. 

A visibilidade corporal constitui um dos aspectos das vivências de pessoas trans, desempenhando papel ambivalente na afirmação identitária e na experiência cotidiana. A condição de serem pessoas facilmente percebidas como diferentes, expõe essas pessoas a situações recorrentes de vigilância e microagressões [26]. Ao mesmo tempo, a necessidade de uma ‘identidade convincente’, sustentada por um corpo que valide esta, pode mobilizar práticas corporais, inclusive a atividade física, como estratégia de construção e afirmação do corpo desejado [20]. Nesse sentido, é possível que a relação observada entre níveis mais elevados de discriminação cotidiana e menor probabilidade de inatividade física reflita trajetórias de engajamento corporal orientadas pela necessidade de reconhecimento social e validação identitária. Estudos prévios [27,28] corroboram essa interpretação ao indicar, que a prática de atividade física entre pessoas trans está associada à necessidade de modificar a aparência física e, por conseguinte, afirmar sua identidade corporal.

Esses achados sugerem que o engajamento em práticas corporais não pode ser compreendido apenas como uma escolha individual, mas como reflexo de determinações históricas, sociais e ambientais que afetam desproporcionalmente grupos vulnerabilizados. É importante evitar que o ônus do sofrimento, advindo da discriminação, recaia sobre o indivíduo e a sua expressão de gênero. O reconhecimento externo de gênero molda a exposição à discriminação e distribui segurança e vulnerabilidade diferenciadamente. Situação essa que reflete um sistema cisnormativo que estrutura espaços e práticas de atividade física a partir de padrões binários de gênero, o que ajuda a produzir barreiras simbólicas e materiais à participação de pessoas trans nesses espaços. 

Revisão bibliográfica revelou que a prática regular de exercícios físicos entre a população trans tem se mostrado essencial para atenuar a violência exercida sobre esses corpos, além de ser importante para a saúde e o bem-estar físico e mental dessa população [28]. Outro aspecto que tem sido problematizado, principalmente entre homens trans, refere-se à possível interrelação entre a prática da atividade física e a construção da sua corporeidade, que perpassam narrativas sociais e imagens que correspondem a uma masculinidade específica [29,30]. O ganho e a definição de massa muscular decorrente da prática de atividade física é, para uma parcela dessa população, parte do processo de masculinização que está sendo buscado, o que contribui para a construção da sua identidade [28].

A inatividade física na população estudada, em síntese, esteve associada positivamente ao menor nível de escolaridade, ao uso de serviço de saúde mental e à baixa qualidade de vida. Por outro lado, as pessoas trans mais ativas tenderam a sofrer maior discriminação cotidiana. Promover a atividade física nessa população demanda, portanto, ações que transcendam a esfera individual para o enfrentamento das iniquidades sociais que atravessam o cotidiano das pessoas trans.

A inatividade física é relevante fator de risco à saúde e potencialmente passível de ser modificado, motivo pelo qual é importante investigá-la em diversos grupos populacionais, como o das pessoas trans. É importante compreender os corpos trans como territórios de disputa e de resistência, que necessitam de intervenções em saúde formuladas a partir de compromisso com a justiça social e com o direito de todos ao bem-estar. 

É premente também o desenvolvimento e o aprimoramento de políticas públicas intersetoriais culturalmente sensíveis, que considerem os determinantes sociais que têm impacto na saúde, a fim de reduzir as barreiras enfrentadas por pessoas trans. Ambientes inclusivos e estratégias específicas de promoção da saúde devem ser implementadas por meio de intervenções intersetoriais, organizadas em rede e orientadas para a equidade.

## Data Availability

O banco de dados e os códigos de análise utilizados na pesquisa estão disponíveis mediante solicitação aos autores.
